# 50 MHz–10 GHz Low-Power Resistive Feedback Current-Reuse Mixer with Inductive Peaking for Cognitive Radio Receiver

**DOI:** 10.1155/2014/683971

**Published:** 2014-07-14

**Authors:** Nandini Vitee, Harikrishnan Ramiah, Wei-Keat Chong, Gim-Heng Tan, Jeevan Kanesan, Ahmed Wasif Reza

**Affiliations:** ^1^Department of Electrical Engineering, University of Malaya, 50603 Kuala Lumpur, Malaysia; ^2^Department of Electrical and Electronic Engineering, Segi University, Jalan Teknologi, Kota Damansara, 47810 Petaling Jaya, Selangor, Malaysia

## Abstract

A low-power wideband mixer is designed and implemented in 0.13 *µ*m standard CMOS technology based on resistive feedback current-reuse (RFCR) configuration for the application of cognitive radio receiver. The proposed RFCR architecture incorporates an inductive peaking technique to compensate for gain roll-off at high frequency while enhancing the bandwidth. A complementary current-reuse technique is used between transconductance and IF stages to boost the conversion gain without additional power consumption by reusing the DC bias current of the LO stage. This downconversion double-balanced mixer exhibits a high and flat conversion gain (CG) of 14.9 ± 1.4 dB and a noise figure (NF) better than 12.8 dB. The maximum input 1-dB compression point (P1dB) and maximum input third-order intercept point (IIP3) are −13.6 dBm and −4.5 dBm, respectively, over the desired frequency ranging from 50 MHz to 10 GHz. The proposed circuit operates down to a supply headroom of 1 V with a low-power consumption of 3.5 mW.

## 1. Introduction

As the number of new wireless applications advents tremendously, the demand for additional frequency spectrum allocation has been growing rapidly. However, the former practice of fixed spectrum allocation policy suffers from the low spectrum utilization setback and this sets a limitation in the available spectrum of accommodating the next generation wireless applications and services [1]. The inefficiency in spectrum usage and the shortage in spectrum have motivated the evolution of CRs. CR was deemed to be an innovative approach due to its versatility in the context of sensing local spectrum reliably and utilizing unoccupied frequency spectrum in the targeted spectral range, while abstaining interference for licensed user by favourably altering its receiving and transmitting parameters [2]. The first international regulation which establishes CRs based system is IEEE 802.22, where the access of unlicensed CR devices on (TV) frequency spectrum (54 MHz–862 MHz) is permitted [3]. Due to excessive demand and technology advancement, the CRs have a greater potential to be expanded further from the constraint of TV band [4].

In the realization of CRs function, a wideband receiver is required to encapsulate the entire aspired frequency band instead of the conventional practice where multiple receivers are envisioned to cover different frequency bands. This highlights the importance of wideband mixer in RF front-end receiver circuit to perform frequency translation. This paper focuses on the design of low-power wideband mixer with a flat and high CG frequency response, along with a flat and low NF frequency response and an adequate linearity over a wide frequency range for CR receiver application. On the platform of CMOS technology, several state-of-the-art architectures for wideband mixer have been reported [5–15]. The double-balanced Gilbert cell mixer has been the mainstay of development in wideband mixer due to its inherent characteristics of high CG and high port-to-port isolation [16, 17]. However, a high CG is inherited at the penalty of high bias current which concurrently contributes towards the flicker noise problem and voltage headroom limitation. In the advantage of minimizing the load resistance in addressing the limitation of voltage headroom, the CG of the mixer is adversely affected. In [18], current-bleeding technique is used to mitigate the output voltage headroom problem by steering the DC current flow through the load resistance. However, this approach is not a preferred solution as the total power consumption remains the same as in conventional Gilbert cell architecture. In addition, the parasitic capacitance introduced by the bleeding transistors not only limits the operating bandwidth, but also degrades the CG by channelling a signal leakage path to the substrate ground.

The well-defined folded-cascode mixer is envisaged to surmount the drawbacks that exist in the Gilbert cell mixer. Instead of stacking the switching stage above the transconductance stage, the folded-switching stage is a promising solution to overcome the voltage headroom limitation. The folded-cascode mixer presented in [19] exhibits a narrow band response and it is not suitable for CR application. Hence, a wideband matching network is integrated at the input mixer to achieve a wideband operating frequency [15]. The mixer input matching circuit not only is crucial as an interstage matching in cascaded systems, but also is essential to ensure the wide bandwidth performance by providing a low input reflection loss across the frequency range.

The RFCR mixer [7, 20] with folded-cascode structure is widely adapted in recent reported work due to the inherent wideband input matching characteristic with low NF and high gain. Although the RFCR architecture had shown good inherent performance but there is severe contradiction in improving wideband input matching and noise due to the large gate-source parasitic capacitance of the input stage transistors. Therefore, a *π*-match LC network is introduced in [21] to enhance the input matching by creating an extra zero while reducing gate noise by series resonance of *L*_*g*_-*C*_in_ at high frequencies. The reported low-voltage, low-power RFCR mixer architecture in [7] achieves a high CG over wide range of frequency bandwidth in 65 nm CMOS technology. As the DC current-reuse is not feasible in a folded architecture, the cost of this implementation would be in increased power consumption relative to the technology of implementation.

The effect of even-order distortion in CRs is more critical than narrow band receivers [4]. This distortion is heavily attributed to the presence of asymmetric mismatch in the downconversion mixer and it causes disturbance to the desired channel. To alleviate this distortion effect, a balanced circuit with differential architecture and symmetrical physical layout is preferred. In this work, a low-voltage, low-power and double-balanced wideband mixer integrates a *π*-match LC network at the input mixer to meet wideband input matching performance. The complementary current-reuse topology [22] improves the power consumption of mixer while enhancing the RF input transconductance. In addition, an inductive peaking is also employed to improve the mixer noise and conversion gain flatness. The proposed mixer is extracted, simulated, and verified on 0.13 *μ*m standard CMOS platform. This paper is organized as follows.  Section 2 reviews and highlights the design limitations and performance trade-offs in conventional RFCR mixer. In Section 3, the circuit topology and operation principles of the proposed mixer are presented. An insight into RFCR mixer operation is given by analyzing the innovative techniques that are adapted to overcome the limitations in confirming the stringent requirements for CR application.  Section 4 reports the RC-extracted postlayout simulation results and Section 5 presents the conclusion.

## 2. Design Challenges

**Figure 1:**
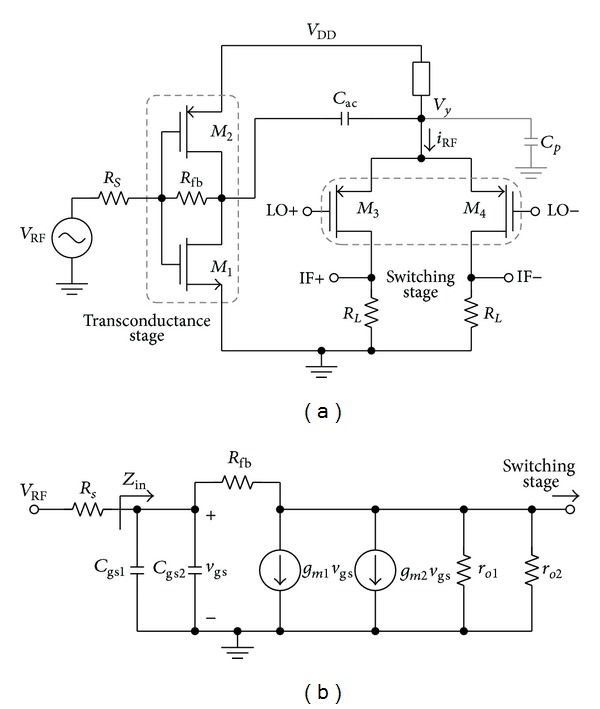
(a) Simplified single-balanced RFCR mixer and (b) small signal equivalent circuit of RFCR transconductance stage.

Several publications were reported on RFCR architecture [7, 20, 23] which can be amenable to both narrow and wideband applications. The RFCR architecture is more viable design for wideband system due to its inherent wideband input matching, low-power consumption, and high gain characteristics. In typical wide bandwidth application, a flat frequency response of gain and NF is preferred across the operating frequency. However, the conventional RFCR architecture tends to suffer from poor NF performance although the CG can be flattened across the range of wide bandwidth due to intrinsic conflict between flat gain and flat NF [21, 24, 25]. Therefore, the conventional RFCR architecture is reviewed in order to analyse the performance parameter trade-offs, which limit the extension of the operational bandwidth. The architecture of Figure 1(a) illustrates the common single-balanced RFCR mixer with the simplified equivalent small signal representation of the transconductance stage given in Figure 1(b). Transistor *M*_1,2_ represents the RF input transistor; transistor *M*_3,4_ is LO switching pair; capacitor *C*_ac_ is the DC-decoupling capacitor; capacitor *C*_*p*_ is the emulation of the parasitic capacitance at node *V*_*y*_ while resistors *R*_*s*_, *R*_fb_, and *R*_*L*(1,2)_ represent the input source resistance, feedback resistor, and load resistor, respectively.

At low frequency, the frequency-dependent component is negligible. From Figure 1(b), the input impedance, *Z*_in_ can be defined as follows:

(1) $$\begin{array}{c} Z_{\text{in}}\approx \frac{R_{\text{fb}}+r_{o}}{1+|A_{vo}|}, \end{array}$$

where *A*_*vo*_ is the open-loop gain, computed as *g*_*m*_ *r*_*o*_, in which *g*_*m*_ is the RF input transconductance represented as *g*_*m*1_ + *g*_*m*2_ and *r*_*o*_ is *r*_*o*1_⫽*r*_*o*2_. Equation (1) reveals that input impedance of the RFCR circuit is mainly determined by resistor *R*_fb_ and transconductance *g*_*m*_. In order to achieve an input reflection coefficient, |Γ | ≤−10 dB, with respect to a source impedance of *R*_*s*_ = 50 Ω, yields *Z*_in_ in a range of 25 Ω to 100 Ω [7, 21].

The voltage gain and noise factor of the transconductance stage can be derived as

(2) $$\begin{array}{c} A_{v}=\frac{r_{o}\left(1-g_{m}R_{\text{fb}}\right)}{R_{\text{fb}}+r_{o}}, \end{array}$$

(3) $$\begin{array}{c} F\approx 1+\frac{R_{\text{fb}}}{R_{s}}{\left(\frac{1+g_{m}R_{s}}{1-g_{m}R_{\text{fb}}}\right)}^{2}+\frac{\gamma g_{m}}{\alpha R_{s}}{\left(\frac{R_{\text{fb}}+R_{s}}{1-g_{m}R_{\text{fb}}}\right)}^{2}, \end{array}$$

where *α* is the ratio between the device transconductance and the zero-bias drain conductance, while *γ* is the channel thermal noise coefficient. From (1) and (3), it can be observed that there is a close relationship between input impedance matching and NF. The NF can be significantly improved by increasing the RF input transconductance and the value of resistor *R*_fb_ at the expense of the input impedance matching performance.

However in practical circuit implementation, the parasitic capacitances introduced by the gate of input transistors further exacerbate the input matching especially at high frequencies. The frequency dependent component is taken into account to finalize the limitation of wideband operating bandwidth; thus (1) can be rewritten as follows:

(4) $$\begin{array}{c} Z_{\text{in}}\left(s\right)=\frac{R_{\text{fb}}+r_{o}}{1+g_{m}r_{o}}\left(\frac{1}{1+s\left(C_{\text{gs}}\left(R_{\text{fb}}+r_{o}\right)/(1+g_{m}r_{o})\right)}\right), \end{array}$$

where *C*_gs_ = *C*_gs1_ + *C*_gs2_. From (4), as *Z*_in_ = *R*_*s*_ = 50 Ω for the perfect matching condition, the maximum gate-source parasitic capacitance can be expressed as follows:

(5) $$\begin{array}{c} C_{\text{gs},\max}=\frac{1}{\pi R_{s}f}\sqrt{\frac{{\left|\Gamma \right|}^{2}}{{\left|\Gamma \right|}^{2}-1}}, \end{array}$$

where *R*_*s*_ represents the input port impedance and *f* is the cut-off frequency. Based on (5), at the targeted input matching of −10 dB with an upper corner frequency of 10 GHz respective to a source impedance of *R*_*s*_ = 50 Ω, the maximum capacitance *C*_gs,max⁡_ which is contributed from both NMOS and PMOS input transistors equals 200 fF. Evidently, a comparative small parasitic capacitance reflects to a small aspect ratio of RF input transistors which concurrently creates limitation in boosting the RF input transconductance and achieving low noise performance.

In addition to this trade-off, the investigation of noise contribution from switching stage reveals more setbacks which further degrade the mixer's performance. The total output noise of a mixer consists of thermal noise and flicker noise. The thermal noise which is mainly dominated by the transconductance stage can be easily reduced by increasing the bias current. The switches in an active mixer predominately contribute towards the growth of flicker noise at the mixer's output. The flicker noise articulated by the LO switches exists at the output of the mixer via direct and indirect mechanism [26]. The flicker noise in effect through the direct mechanism is due to random modulation of the duty cycle of the output current, whereas through the indirect mechanism it is caused by charging and discharging of the parasitic capacitances between transconductance and LO stages. The output noise current generated by the direct mechanism and indirect mechanism is given as in the following [26, 27]:

(6) $$\begin{array}{c} i_{o,n(\text{direct})}=\frac{4I_{\text{tail},\text{sw}}V_{n}}{ST}, \end{array}$$

(7) $$\begin{array}{c} i_{o,n(\text{indirect})}=\frac{2C_{p}}{T}V_{n}\frac{{\left(C_{p}\omega_{\text{LO}}\right)}^{2}}{{\left(g_{m,\text{sw}}\right)}^{2}+{\left(C_{p}\omega_{\text{LO}}\right)}^{2}}, \end{array}$$

where *I*_tail,sw_ is the DC tail current in switching stage, *V*_*n*_ is the equivalent flicker noise of the switching pair, *S* is the slope of LO signal, *T* is the LO period, *C*_*p*_ is the parasitic capacitance at switching tail, *ω*_LO_ is the frequency of LO signal, and *g*_*m*,sw_ is the transconductance of switching transistor. In reference to (6) and (7), the direct and indirect noise currents are evidently proportional to the flicker noise voltage, *V*_*n*_ of LO transistor which is expressed as in the following:

(8) $$\begin{array}{c} V_{n}=\sqrt{\frac{2K_{f}}{W_{\text{eff}}L_{\text{eff}}C_{\text{ox}}f}}, \end{array}$$

where *K*_*f*_ is the technology parameter, *W*_eff_ and *L*_eff_ are the effective width and length of LO transistor, respectively, *C*_ox_ is the oxide capacitance of LO transistor, and *f* is the operating frequency. Apparently, in order to minimize the flicker noise effect caused by the direct mechanism, low DC current at the switching stage and large size of LO transistor are preferred. On the contrary, the larger size of LO switching transistor yields to a larger parasitic capacitance, *C*_*p*_ at node *V*_*y*_ as referred to in Figure 1(a). This results in an increase of noise current from indirect mechanism as can be observed from (7). In addition, the capacitance *C*_*p*_ also creates detrimental effect at high frequency by introducing a low impedance path for RF signal which shunts the RF signal to the ground, thus reducing the CG and adversely limiting the operational bandwidth. This effect can be mathematically proven by deriving the pole frequency of the effective transconductance of the mixer in Figure 1(a) as in the following:

(9) $$\begin{array}{c} \frac{i_{\text{RF}}\left(s_{\text{RF}}\right)}{v_{\text{gs}}\left(s_{\text{RF}}\right)}=\frac{1-g_{m}R_{\text{fb}}}{R_{\text{fb}}+R_{\text{eq}}}\left(\frac{1}{s_{\text{RF}}C_{p}\left(R_{\text{eq}}⫽R_{\text{fb}}\right)+1}\right), \end{array}$$

where *R*_eq_ is total resistance at the node *V*_*y*_. Hence, the pole frequency of the mixer which plays a crucial role in determining the operating bandwidth of the mixer core can be derived as

(10) $$\begin{array}{c} \omega_{\text{RF}}=\frac{1}{C_{p}\left(R_{\text{eq}}⫽R_{\text{fb}}\right)}. \end{array}$$

It is noted that the capacitor *C*_*p*_ forms as a low-pass filter at the tail of switching quad, where the gain response rolls off beyond the cut-off frequency. Therefore, it can be concluded that obtaining a large operation bandwidth with relatively large capacitor *C*_*p*_ at node *V*_*y*_ is not feasible. This has driven the need for the exploration of new design technique to achieve large bandwidth performance.

## 3. Proposed Mixer

**Figure 2:**
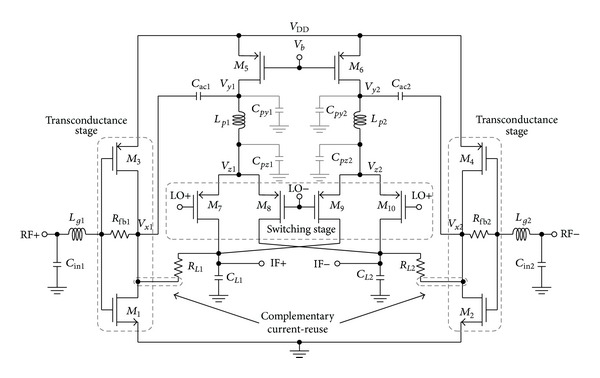
Proposed wideband mixer.

The proposed RFCR mixer illustrated in Figure 2 consists of RF input transistors *M*_1,2_, current-reuse PMOS bias transistors *M*_3,4_, feedback resistor *R*_fb(1,2)_, DC-decoupling capacitor *C*_ac(1,2)_, switching transistors *M*_7_–*M*_10_, peaking inductor *L*_*p*(1,2)_, and passive load of *R*_*L*(1,2)_ and *C*_*L*(1,2)_. The folded architecture is preferred over the conventional series stacking topology due to its merit in low voltage headroom realization. The minimum voltage headroom that can be applied to the designed circuit is approximated as

(11) $$\begin{array}{c} V_{\text{DD},\min}=V_{\text{ds}1,2(\mathrm{sat})}+V_{\text{ds}3,4(\mathrm{sat})}+V_{\text{th},n}+V_{\text{th},p}, \end{array}$$

where *V*_ds1,2(sat⁡)_ and *V*_ds3,4(sat⁡)_ are the overdrive voltage of transistors *M*_1,2_ and *M*_3,4_, respectively, while *V*_th,*n*_ and *V*_th,*p*_ are the respective threshold voltage of the transistors *M*_1,2_ and *M*_3,4_.

The transconductance stage is realized through the integration of inverter with feedback resistor, *R*_fb(1,2)_. At the transconductance stage, PMOS transistor *M*_3,4_ is stacked at the top of NMOS transistor *M*_1,2_ to form a current-reuse topology. Therefore, the transistor *M*_3,4_ enhances the RF input transconductance *g*_*m*,RF_ to *g*_*m*(1,2)_ + *g*_*m*(3,4)_ without additional power consumption compared to a single N-type common source amplifier associated with an RF input transconductance, *g*_*m*,RF_ = *g*_*m*(1,2)_. In addition, the PMOS transistor *M*_3,4_ also provides high intrinsic output impedance to prevent RF signal leakage to the power supply. The resistor *R*_fb(1,2)_ is used not only to meet the aspired input impedance matching criterion, but also to reduce the power consumption in line to the elimination of additional biasing circuitry for transistors *M*_1_–*M*_4_ in the context of self-biased principle. By adapting the complementary current-reuse technique, the DC current from the switching stage is fed into transistor *M*_1,2_ instead of being routed into silicon ground as in a typical folded topology apparently in a quest to boost the gain without additional power consumption. As a result, the NMOS transistor *M*_1,2_ contributes more transconductance than PMOS transistor *M*_3,4_ due to an increased current flow through the transistor *M*_1,2_; thus the aspect ratio of transistors *M*_1,2_ and *M*_3,4_ along with feedback resistor *R*_fb(1,2)_ is optimized diligently according to (1) and (3).

As mentioned before, a large aspect ratio of RF input transistors contributes to a respective large gate-source parasitic capacitance at the input stage of the conventional RFCR topology and thus adversely affects the input matching and concurrently reduces the operating bandwidth. Hence, an inductor *L*_*g*(1,2)_ is placed in series with the gate of the transconductance stage transistors while the input capacitor *C*_in(1,2)_ is placed in parallel to the transconductance stage to extend the input bandwidth of the frequency response in achieving a good matching over the operating frequency range. The capacitor *C*_in(1,2)_ and inductor *L*_*g*(1,2)_ integrated with the total gate-source capacitance, *C*_gst_ of input transistors, form a third-order LC ladder low-pass filter. In this approach, the inductor *L*_*g*(1,2)_ coupled with the capacitor *C*_in(1,2)_ to eliminate the effect of *C*_gst_ and to resonate out the reactive component of *Z*_in_ at the desired frequency. Through this technique, the constraints of gate-source parasitic capacitance as described in (5) are relaxed. In preference the transconductance can be increased to achieve higher gain and low noise performance simultaneously by increasing the size of RF input transistor while retaining the operating bandwidth as there is an additional degree of freedom in increasing *C*_gst_.

**Figure 3:**
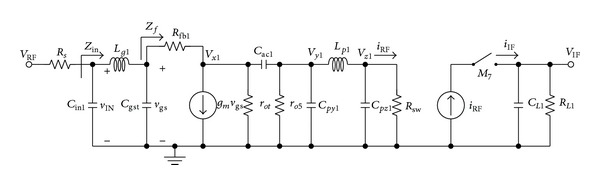
Half circuit small signal representation of the proposed wideband mixer.

Figure 3 depicts the corresponding half circuit small signal representation of the proposed wideband mixer which is illustrated in Figure 2. The capacitors *C*_*py*(1,2)_ and *C*_*pz*(1,2)_ represent the parasitic capacitances at nodes *V*_*y*(1,2)_ and *V*_*z*(1,2)_, respectively. To simplify the analysis, the DC blocking capacitor *C*_ac(1,2)_ between the transconductance and switching stage is neglected since the impedance in effect of *C*_ac(1,2)_ is relatively small at the operating frequency range. The input impedance *Z*_in_ and the input return loss *S*_11_ of the proposed architecture can be derived and expressed as in (12) and (13), respectively,

(12) $$\begin{array}{c} Z_{\text{in}}=\frac{s^{2}C_{\text{gst}}L_{g1}Z_{f}+sL_{g1}+Z_{f}}{s^{3}C_{\text{gst}}C_{\text{in}1}L_{g1}Z_{f}+s^{2}C_{\text{in}1}L_{g1}+sZ_{f}\left(C_{\text{gst}}+C_{\text{in}1}\right)+1}, \end{array}$$

(13) |S11|=|(−s3CgstCin1Lg1ZfRs+s2CgstLg1Zf −s2Cin1Lg1Rs+sLg1 −sZfRs(Cgst+Cin1)+Zf−Rs)×(s3CgstCin1Lg1ZfRs+s2CgstLg1Zf  +s2Cin1Lg1Rs+sLg1  +sZfRs(Cgst+Cin1)+Zf+Rs)−1|,

where *C*_gst_ = *C*_gs1_ + *C*_gs3_ is total gate-source capacitance of the input transistors and *Z*_*f*_ denotes the impedance looking into the resistor *R*_*fb*1_. Assume that the *π*-match LC network is symmetrical for perfect input impedance matching by equating the capacitor *C*_in(1,2)_ to capacitor *C*_gst_ and *Z*_in_ = *Z*_*f*_ which is typically 50 Ω. From (12), a good input matching for this circuit is obtained at frequencies

(14) ωo1=0,ωo2=2CgstLg1−1Cgst2Zf2.

As can be seen from (14), the two frequencies, *ω*_*o*1_ and *ω*_*o*2_, are adjusted to be located at DC and high frequency, respectively. The frequency *ω*_*o*2_ is optimized to be in the vicinity of frequency *ω*_*o*1_ in order to maintain an input reflection of *S*_11_ below −10 dB across the entire operating frequency confirming a good input matching response inherited. At node *V*_*x*(1,2)_ of Figure 2, the resistive load, *R*_*L*(1,2)_, is designed to be relatively large compared to the impedance looking into the switching transistors; thus the RF signal is driven to subsequent stage through the AC coupling capacitor, *C*_ac(1,2)_. In the worst case scenario, a small amount of RF signal leakage through the load resistor *R*_*L*(1,2)_ can still be shorted out to ground through the load capacitor *C*_*L*(1,2)_ instead of being routed to the IF output. Since the impedance looking into transistor *M*_5,6_ at node *V*_*y*(1,2)_ is also large, the RF signal is forced to enter the switching quad.

A PMOS based local oscillator (LO) switching stage is adopted in place of conventional NMOS transistor as PMOS transistor inherits an intrinsic characteristic of low flicker noise performance and less LO power sensitivity compared to NMOS transistor [23]. In reference to (6), large switching transistors with low LO current are applied to minimize the flicker noise in the direct mechanism. In contrast, a large switching transistor indirectly translates flicker noise to the mixer output due to the presence of large tail capacitance at the switches as described in the previous section. The inherited parasitic capacitance also limits the bandwidth, hence promoting the exploration of inductive peaking technique as illustrated in Figure 2.

The inductor *L*_*p*(1,2)_ is placed at the tail of the switches to enhance the bandwidth through a peaking at high frequency without consuming additional power and voltage headroom. The aspect ratio of transistors *M*_5,6_ and switching transistors are selected appropriately to form two suitable parasitic capacitors *C*_*py*(1,2)_ and *C*_*pz*(1,2)_ at nodes *V*_*y*(1,2)_ and *V*_*z*(1,2)_, respectively. These capacitors form a virtual *π*-network along with inductor *L*_*p*(1,2)_, relaxing the requirement of integrating additional capacitors which degrade the CG and NF. From the perspective of transient analysis, the current charges the two capacitors *C*_*p*(*y*,*z*)_ separately through the inductor *L*_*p*(1,2)_, at different point of time, resulting in the charging time to be reduced leading to an enhancement in bandwidth. In further analyzing the operation of the peaking inductor *L*_*p*(1,2)_, in reference to the frequency response, the LO transistor is modelled as an ON-OFF switch [28] while the resistor *R*_sw_ represents the resistance at the source terminal of switching transistor as illustrated in Figure 3. Based on this approximated model, the overall conversion gain of the mixer is computed by the following expression:

(15) $$\begin{array}{c} A_{v}=\frac{v_{\text{IF}}\left(s_{\text{IF}}\right)}{v_{\text{IN}}\left(s_{\text{RF}}\right)}=\frac{v_{\text{IF}}\left(s_{\text{IF}}\right)}{i_{\text{IF}}\left(s_{\text{IF}}\right)}\cdot \frac{i_{\text{IF}}\left(s_{\text{IF}}\right)}{i_{\text{RF}}\left(s_{\text{RF}}\right)}\cdot \frac{i_{\text{RF}}\left(s_{\text{RF}}\right)}{v_{\text{gs}}\left(s_{\text{RF}}\right)}\cdot \frac{v_{\text{gs}}\left(s_{\text{RF}}\right)}{v_{\text{IN}}\left(s_{\text{RF}}\right)}, \end{array}$$

where *s*_RF_ = *jω*_RF_ is the RF input frequency and *s*_IF_ = *jω*_IF_ is the IF output frequency.

The transfer function of [*v*_gs_(*s*_RF_)/*v*_IN_(*s*_RF_)] and [*v*_IF_(*s*_IF_)/*i*_IF_(*s*_IF_)] can be solved by small signal analysis which are given by (16) and (17), respectively, while the transfer function of [*i*_IF_(*s*_IF_)/*i*_RF_(*s*_RF_)] can be derived adapting Fourier series analysis by approximating the LO signal as an ideal square wave, which is given by (18)

(16) vgs(sRF)vIN(sRF)=ZfsRF2CgstLg1Zf+sRFLg1+Zf,

(17) vIF(sIF)iIF(sIF)=RL11+sIFCL1RL1,

(18) iIF(sIF)iRF(sRF)=2π.

Since the impedances looking through the transistors *M*_1,3_ at node *V*_*x*1_ and transistor *M*_5_ at node *V*_*y*1_ are relatively large, hence the intrinsic resistances *r*_*ot*_ = *r*_*o*1_⫽*r*_*o*3_ and *r*_*o*5_ in Figure 3 are neglected. With the integration of the peaking inductor, the frequency response of the RF signal in (9) is computed as in (19). The transfer function in (19) can be rewritten in expressing a single real pole and two complex poles as follows:

(19) iRF(sRF)vgs(sRF) =(1−gmRfb1Rfb1+Rsw)  ×(sRF3[Cpy1Cpz1Lp1Rfb1RswRfb1+Rsw]    +sRF2[Lp1(Cpy1Rfb1+Cpz1Rsw)Rfb1+Rsw]    +sRF[Rfb1Rsw(Cpy1+Cpz1)+Lp1Rfb1+Rsw]+1)−1,

(20) $$\begin{array}{c} \frac{i_{\text{RF}}\left(s_{\text{RF}}\right)}{v_{\text{gs}}\left(s_{\text{RF}}\right)}=\frac{\left(\left(1-g_{m}R_{\text{fb}1}\right)/\left(R_{\text{fb}1}+R_{\text{sw}}\right)\right)}{\left(1+s_{\text{RF}}/\omega_{0}\right)\left(1+s_{\text{RF}}/Q\omega_{1}+s_{\text{RF}}^{2}/\omega_{1}^{2}\right)}. \end{array}$$

Comparing (19) and (20), the pole factor *Q*, the real pole frequency *ω*_0_, and the complex pole frequencies *ω*_1_ can be expressed as follows:

(21) Q=1×(ω1((Rfb1⫽Rsw)(Cpy1+Cpz1)    +Lp1Rfb1+Rsw−1ω0))−1,

(22) ω0=1ω12Cpy1Cpz1Lp1(Rfb1⫽Rsw),

(23) ω1≈Rfb1+Rsw2QwoLp1(Cpy1Rfb1+Cpz1Rsw)·(1+1+4Q2ωo2Lp1(Cpy1Rfb1+Cpz1Rsw)Rfb1+Rsw).

Notably, the bandwidth extension is heavily dependent on the value of parasitic capacitances *C*_*py*(1,2)_ and *C*_*pz*(1,2)_, inductor *L*_*p*(1,2)_, and resistors *R*_sw_ and *R*_fb(1,2)_. The real pole results in gain and bandwidth reduction at the frequency higher than its value, whereas the complex poles can be adjusted to provide a peaking in frequency response which compensates this adverse effect. Since the real pole is the dominant parameter in achieving a high bandwidth and gain, it should be peaked at the highest frequency as possible, while the location of complex poles are adjusted accordingly to compensate for the gain drop at high frequencies by introducing a peaking and further extending the bandwidth. However, bandwidth enhancement using this approach introduces in-band ripples. Increasing the *Q* potentially enhances the gain at the peaking; however increasing *Q* excessively would result in bandwidth reduction. Similarly increasing *ω*_1_ shifts the peaking to higher frequencies; however when *ω*_1_ is increased excessively, it results in the reduction of gain in reference to (21) and (22). Therefore, *ω*_1_ and *Q* are optimized appropriately to obtain relatively flat and high gain response over the wide bandwidth of operation.

The mixing point of RF and LO signals is located at the node *V*_*z*(1,2)_. The switching quad *M*_7_–*M*_10_ is biased in the vicinity of the threshold voltage at low bias current, thus reducing the DC offset and flicker noise while resulting in a substantial increase in switching efficiency. The low bias current allows the integration of larger load resistance, thus increasing the CG of the mixer and relaxing the constraint of voltage headroom consumption. Load capacitor *C*_*L*(1,2)_ couples with the load resistor *R*_*L*(1,2)_ presenting a low-pass filter at the IF output with the output real pole equal to *ω*_IF_ = 1/*R*_*L*_ *C*_*L*_ based on (17). This integration suppresses the feed components of sin(*ω*_LO_)*t*, sin(*ω*_RF_)*t*, and other unwanted harmonics including the higher-order mixing spurs such as sin(*mω*_LO_ ± *nω*_RF_)*t*, where *m* and *n* are integers. Ultimately, the overall conversion gain of the presented wideband mixer at the desired output spectrum is given by

(24) CG=2π·ZfsRF2CgstLg1Zf+sRFLg1+Zf·(1−gmRfb1)×(sRF3Cpy1Cpz1Lp1Rfb1Rsw  +sRF2Lp1(Cpy1Rfb1+Cpz1Rsw)  +sRF[Rfb1Rsw(Cpy1+Cpz1)+Lp1]  +Rfb1+Rsw)−1·RL11+sIFCL1RL1·[sin⁡(ωLO−ωRF)t].

## 4. RC-Extracted Simulation Results

**Figure 4:**
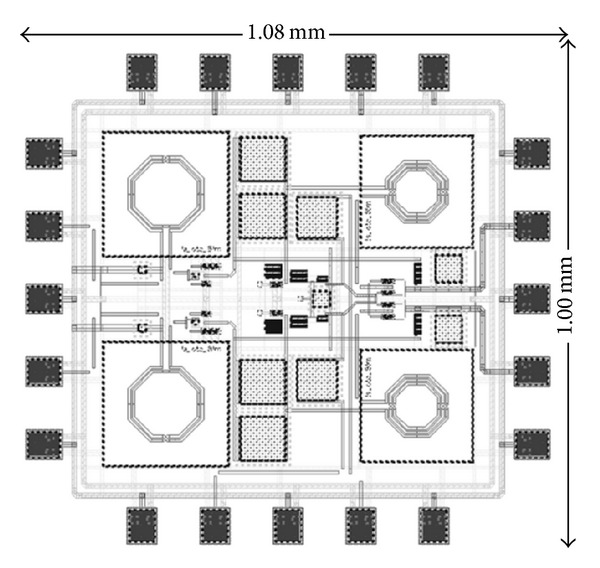
Physical layout of wideband mixer including RF ESD pads.

The proposed wideband mixer of Figure 2 has been designed and simulated using 0.13 *μ*m CMOS standard process for regulated CR applications. The layout parasitic extraction (LPE) is executed and validated under Cadence Spectre-RF and Mentor Calibre platform. In an interest of perfect matching and the minimization of mismatch parasitic coupling effect, the components and metal paths in the designed mixer circuit were placed as symmetrical as possible. The physical layout of the circuit including the RF ESD pads is illustrated in Figure 4 with a total chip area consumption of 1.08 × 1.00 mm^2^.

**Figure 5:**
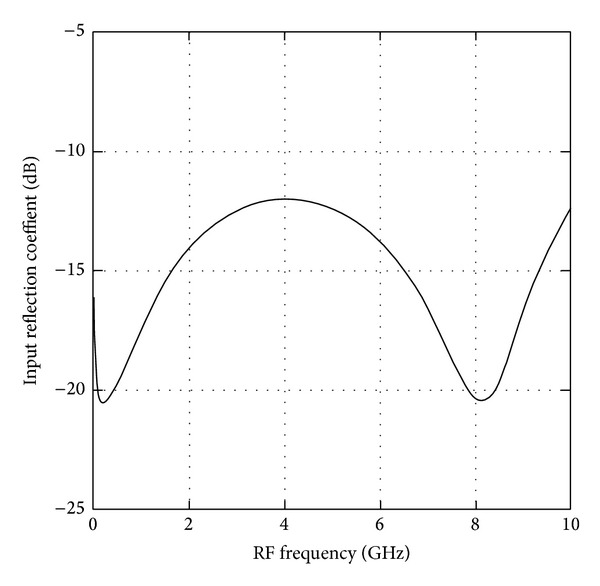
Simulated result of input return loss (*S*_11_).

The postlayout simulation results were carried out with a total power consumption of 3.5 mW at respective voltage headroom of 1 V. The RF input of the wideband mixer is matched to 50 Ω termination and the respective simulated input return loss, *S*_11_, is illustrated in Figure 5. The *S*_11_ of the optimized RFCR wideband mixer is achieved well below −12 dB across the operating frequency ranging from 50 MHz to 10 GHz.

**Figure 6:**
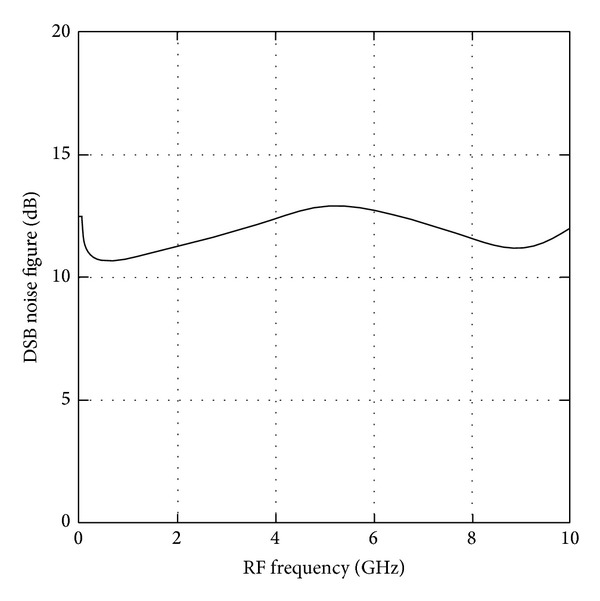
Simulated NF versus RF frequency.

**Figure 7:**
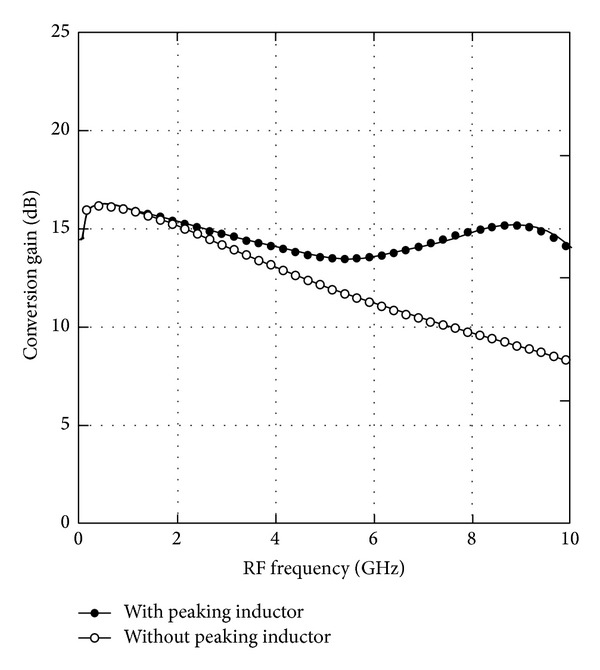
Simulated CG versus RF frequency.

Figure 6 shows the simulated NF versus RF frequency from 50 MHz to 10 GHz with a fixed IF output at 10 MHz while the LO power is set to be 0 dBm. The simulated minimum and maximum NF of the wideband mixer are 10.8 dB and 12.8 dB, respectively. This wideband mixer exhibits a flat NF with a variation of ±1 dB across the entire frequency range.  Figure 7 shows the simulated CG versus RF frequency in a comparison plot with the presence of the peaking inductor and absence of the peaking inductor. At low frequency, the CG is observed to be around 16 dB. However, at high frequency range, the CG is achieved to about 8 dB without the peaking inductor in place and about 14 dB with the integration of peaking inductor, resulting in 6 dB of gain improvement. This plot reveals and confirms that the peaking inductor in RFCR mixer had improved the CG at high frequency range. The proposed wideband mixer achieves a high gain with a flatness variation of ±1.4 dB where the maximum CG of 16.3 dB is observed at 500 MHz and a minimum of CG of 13.5 dB is observed at 5.5 GHz.

**Figure 8:**
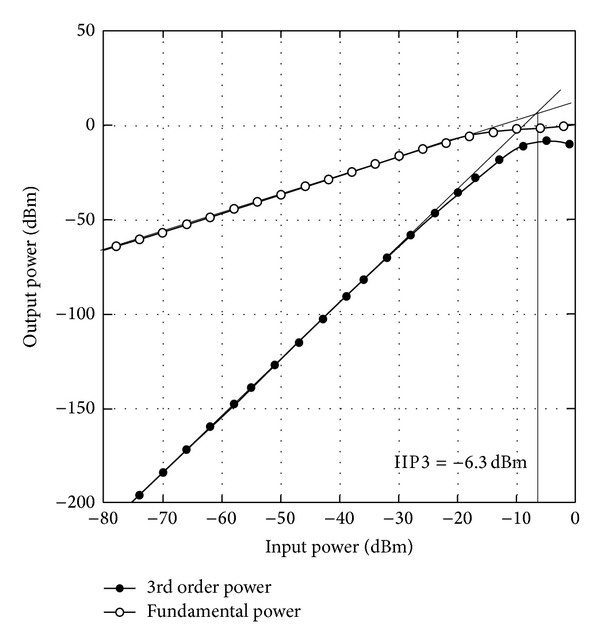
Two-tone IIP3 simulation at 5 GHz.

**Figure 9:**
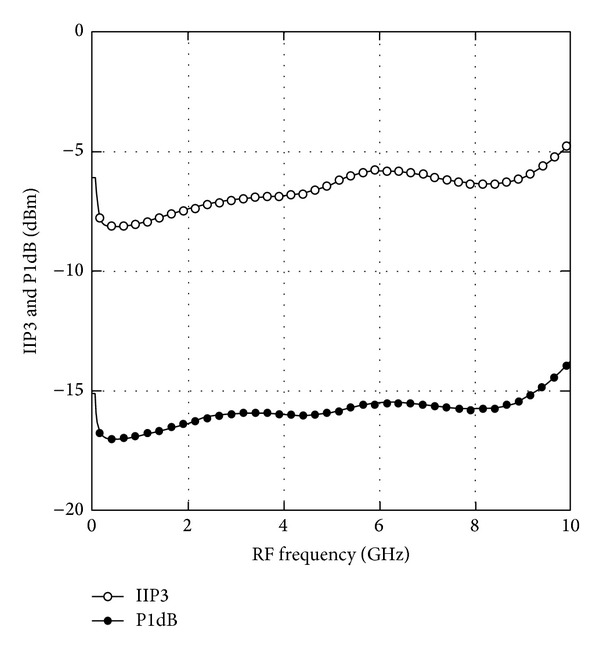
Simulated IIP3 and P1dB versus RF frequency.

In observing the linearity response, the center frequency of 5 GHz from the operating bandwidth is selected. With an LO power of 0 dBm at *ω*_LO_ = *ω*_RF_ + 10 MHz, the P1dB is simulated to be −15.8 dBm. Applying two-tone test with 1 MHz frequency offset, the simulated IIP3 is −6.3 dBm as shown in Figure 8.  Figure 9 depicts the overall performance of the simulated P1dB and IIP3 against RF frequency of the mixer over the range of 50 MHz to 10 GHz. The mixer achieves a P1dB range of −17.0 dBm to −13.6 dBm while the IIP3 ranges from −8.1 dBm to −4.5 dBm.

The overall performance of the proposed mixer can be weighed comparatively with other reported works using a figure-of-merits (FOM). Generally, the mixer performance was compared in terms of CG, NF, linearity (IIP3 or input P1dB), and power consumption [29, 30]. However, a trade-off exits between power dissipation and bandwidth in wideband mixer design. Hence, it is essential to include the operating bandwidth parameter into FOM calculation for the fair comparison with the reported works [31]. As a result, a modified FOM is introduced for wideband mixer, which is given as

(25) $$\begin{array}{c} \text{FOM}=10\log\left(\frac{10^{\text{C}{\text{G}}_{\max}/20}\cdot 10^{\left(\text{IIP}3_{\max}-10\right)/20}}{10^{\text{N}{\text{F}}_{\min}/10}\cdot P_{d}}\cdot \frac{f_{H}-f_{L}}{\sqrt{f_{H}f_{L}}}\right), \end{array}$$

where *f*_*L*_ and *f*_*H*_ represent lower cut-off frequency and upper cut-off frequency, respectively. *P*_*d*_ is the power consumption in Watts. CG_max⁡_ is the maximum conversion gain, IIP3_max⁡_ is the maximum input third-order intercept point, and NF_min⁡_ is the minimum noise figure. The simulated results of the proposed architecture along with other reported results of the recent works are tabulated in Table 1. The proposed mixer achieves 26.14 dB which is the highest FOM compared to other mixers.

## 5. Conclusion

In this work, a new wideband mixer for CR receiver has been successfully designed and simulated in 0.13 *μ*m CMOS process. A *π*-match LC network is embedded at the input of RFCR architecture to simultaneously enhance the input impedance matching and NF while encapsulating an operating bandwidth as large as 10 GHz. The RFCR adaptation enables the proposed mixer to achieve high gain by summing up the transconductance of NMOS and PMOS in the transconductance stage. The peaking inductor achieves a flat CG response by compensating the gain degradation at high frequencies, while extending the bandwidth. Additionally, the complementary current-reuse technique is implemented at the output stage to further boost the CG without dissipating additional power. The proposed wideband mixer operates from 50 MHz to 10 GHz with an RF input return loss better than −12 dB, a high CG of 14.9 ± 1.4 dB, a flat NF of 11.8 ± 1 dB, an P1dB of −15.3 ± 1.7 dBm, and an IIP3 of −6.3 ± 1.8 dBm. This mixer operates at a low voltage headroom of 1.0 V while consuming only 3.5 mW of power. This characteristic of proposed wideband mixer serves to be a compatible architecture to meet the future growing demands in CR application.

## Figures and Tables

**Table 1:** Summary of wideband mixer performance and comparison with prior published work.

Reference	Process (*µ*m)	Freq. (GHz)	CG (dB)	NF (dB)	P1dB (dBm)	IIP3 (dBm)	*V*_DD_ (V)	*P*_DC_ (mW)	FOM (dB)
[5]^a^	0.18	3.4~6.8	5.75 ± 1.45	14.15 ± 0.25	—	2.5 ± 0.5	1.0	2.9	10.07
[6]^a^	0.13	0.87~3.7	13.75 ± 0.25	4.6 ± 1.9	—	−11.5 ± 1.5	1.2	16.8	14.03
[7]^a^	0.065	1~10.5	10.15 ± 2.65^c^	9.8 ± 2.2^c^	−12.7 ± 2.3^c^	−3.5 ± 3.5^c^	1.0	5.0	21.48
[8]^a^	0.13	3.1~10.6	11.9 ± 2.10	17.05 ± 2.55	−21.5 ± 2.5	−13.5 ± 2.5^c^	1.2	1.85	10.5
[9]^a^	0.18	0.5~7.5	4.35 ± 1.35^c^	15	−16	−11	0.77	0.48	18.77
[10]^a^	0.18	2~11	6.9 ± 1.5	17.75 ± 2.25^c^	−6 ± 2.5^c^	4.25 ± 2.25^c^	1.8	25.7	5.68
[11]^a^	0.045	1~10	8.35 ± 1.05	21.2	−14.6 ± 1.4	—	1.1	1.46	—
[12]^a^	0.13	1~10	5.5 ± 2.5	13.15 ± 1.85	14.5 ± 1.5^c^	−5.5 ± 1.5	1.2	8.4	11.0
[13]^a^	0.18	2.3~5.8	2.26 ± 1.48	20.5 ± 1.3	−17.5 ± 2.5	6.05 ± 1.35	1.8	8.3	2.0
[14]^b^	0.13	3.1~4.8	11.25 ± 1.25^c^	12.75 ± 0.25^c^	—	24	1.2	3.0	24.92
[15]^a^	0.18	0.2~16	7.0 ± 1.7^c^	—	—	—	1.8	15	—
This work	0.13	0.05~10	14.9 ± 1.4	11.8 ± 1	−15.3 ± 1.7	−6.3 ± 1.8	1.0	3.5	26.14

^a^Measured results.

^b^Simulated results.

^c^Estimated value.
